# Investigation of the Thermal, Physical, and Microstructural Properties of Polymeric Composites Bio-Reinforced with Charcoal Fines

**DOI:** 10.3390/polym17101370

**Published:** 2025-05-16

**Authors:** Josinaldo O. Dias, Amanda O. Conceição, Rayara Siqueira, Bruno Fonseca Coelho, Patrícia S. Oliveira

**Affiliations:** 1 Agricultural Sciences and Engineering Center, Federal University of Espírito Santo, Vitoria 29075-910, Brazil; amanda.o.conceicao@edu.ufes.br (A.O.C.); rayara.siqueira@edu.ufes (R.S.); bruno.coelho@edu.ufes.br (B.F.C.); 2 Federal Center for Technological Education of Minas Gerais, Belo Horizonte 30421-169, Brazil; patricia@cefetmg.br

**Keywords:** composite materials, HDPE, thermogravimetric analysis (TGA), Fourier transform infrared spectroscopy (FTIR), X-ray diffraction (XRD)

## Abstract

Incorporating solid waste into polymeric matrices has proven effective in developing composites with enhanced mechanical and thermal properties. This study investigates a composite based on recycled high-density polyethylene (HDPE), reinforced with fine charcoal particles, assessing its thermal, microstructural, and density properties. Two processing methods (compression molding and extrusion) and four charcoal concentrations (0%, 5%, 10%, and 15 wt%) were evaluated. Thermal characterization was performed using thermogravimetric analysis (TGA) and Fourier transform infrared spectroscopy (FTIR). The microstructure was analyzed through scanning electron microscopy (SEM) and X-ray diffraction (XRD), while the density was determined via X-ray densitometry. SEM revealed increased porosity with charcoal addition. The thermal properties and crystallinity of the composites were not significantly affected by variations in the manufacturing method or charcoal concentration. FTIR analysis identified characteristic peaks, while TGA indicated mass loss between 400 and 500 °C, with a maximum decomposition temperature of 487 °C. XRD confirmed the semicrystalline structure typical of HDPE. Thus, incorporating charcoal residues can reduce the use of fossil-based materials while providing a sustainable application for industrial waste.

## 1. Introduction

Polyethylene (PE) is the most widely used polymer globally [1], valued for its intrinsic properties, including chemical stability and versatility. These characteristics, combined with its durability, ensure PE’s prominent presence across various sectors, such as construction, packaging, and the automotive industry [2]. However, the polymer production remains heavily reliant on fossil resources and has significantly contributed to increasing pressure on these resources and exacerbating environmental issues associated with plastic production and disposal. The persistence of these materials in the environment, coupled with their large-scale production, has intensified regulatory pressure, aligning with the growing demands of industries and consumers worldwide [3]. This scenario has driven manufacturers and researchers to seek sustainable and environmentally friendly alternatives to replace conventional plastics and reduce dependence on petroleum-derived resources [4].

The quest for polymeric materials with enhanced properties for specific applications has driven the development of various modification strategies [5,6,7]. One widely used physical modification method is the incorporation of specific reinforcements [8]. This approach enables the production of composites with enhanced thermal properties due to the synergy between the intrinsic properties of the components [9,10].

The incorporation of solid waste into polymeric matrices has proven to be an effective strategy for developing composites with improved mechanical and thermal properties, such as increased modulus of elasticity, glass transition temperature, and heat deflection temperature [1]. However, the final properties of the composite are strongly influenced by several factors, including the nature of the reinforcement (origin, structure, and particle size); the composition of the polymeric matrix; and the processing conditions [11].

The construction of compatible interfaces between reinforcements and the matrix plays a crucial role in the preparation of composites with superior properties. The lipophilic and nonpolar nature of the carbonaceous fraction present in fine charcoal particles enhances the compatibility with polymeric matrices, which also tend to be nonpolar, during melt processing. This promotes improved interfacial adhesion and, consequently, more effective reinforcement [12].

In this context, the present study aimed to characterize the thermal and microstructural properties of high-density polyethylene (HDPE)-based composites and fine charcoal particles. The composites were produced using compression molding and extrusion techniques. Thermal characterization was performed using thermogravimetric analysis (TGA) and Fourier transform infrared spectroscopy (FTIR). The microstructure was analyzed through scanning electron microscopy (SEM) and X-ray diffraction (XRD), while the density was determined via X-ray densitometry.

## 2. Materials and Methods

For charcoal production, wood residues were used from *Eucalyptus* sp. The wood samples were initially ground in a Tigre A4 hammer mill (Moinhos Tigre, São Paulo/Brazil) for 2 min per 500 g of biomass. Then, they were processed in a Willy TE-650 knife mill (Tecnal, Piracicaba/BRA), achieving a sawdust particle size of approximately 50 mesh. This step is essential, as the smaller particle size of the biomass enhances heat transfer during pyrolysis, leading to a higher yield of fine charcoal particles. The ground wood samples were dried in an oven at 102 ± 2 °C for 20 h. The dried material was then pyrolyzed in an electric muffle furnace at a final temperature of 800 °C, with a heating rate of 5 °C min^−1^ and a residence time at the final temperature of 60 min. These procedures followed the descriptions of [13].

### 2.1. Production of the Composites

The recycled HDPE was obtained through a partnership with the Association of Recyclable Material Collectors and collection efforts carried out in communities in the city of Jerônimo Monteiro, ES. Product packaging was collected and cleaned with water and neutral detergent to remove residues, impurities, and contaminants. The cleaned packaging was manually cut into smaller pieces using standard scissors. The cut pieces were then ground in a knife mill to reduce the particle size to 18 mesh (1.00 mm).

To assess the impact of different production methods and the incorporation of charcoal fines into the polymeric structure, two manufacturing processes were defined, and specific fine proportions were established for the experimental design (Table 1).

The composites were produced at the Department of Chemistry (DEQUI) of CEFET-MG, located in Belo Horizonte, Minas Gerais, through thermal processing using pressing and extrusion methodologies. For both methods, material preparation was conducted to ensure better homogenization of the matrix and bio-reinforcement. The samples were prepared in an M.H. Equipment homogenizer, model MH-100 (MH, Guarulhos, Brazil). The mixture was maintained in the chamber for 25 s under a maximum rotation of 100 rpm. After this step, the material was granulated in a KIE knife mill, model MAC250BX (KIE, Campinas, Brazil), for about 1 min. Finally, the process of producing the composite materials was initiated.

**Figure 1:**
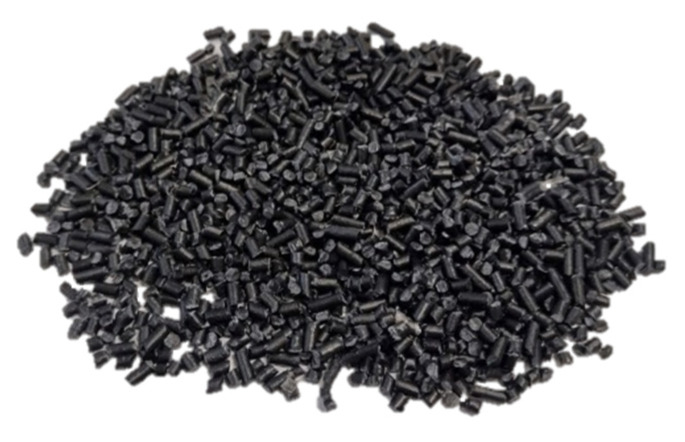
Pelleted material.

A single-screw extruder from THERMO SCIENTIFIC, model HAAKE POLYLAB QC (Thermo Fisher, Waltham, MA, USA), with four heating zones, was used for the extrusion process. The homogenized and mixed sample was fed into the extruder using the following parameters: (i) screw rotation speed of 20 rpm and (ii) temperature profile set to 160 °C for heating zones 1, 2, and 3 and (iii) 170 °C for zone 4 (exit or die zone). After processing, the filaments were pelletized (Figure 1) using an AXPlástico Granulator, Model AX Gran (AXPlástico, Diadema, Brazil).

The pelletized material was pressed into sheets approximately 150.0 × 100.0 × 1.4 mm in size using a hydraulic press with heating, model SL11 by SOLAB (SOLAB, Piracicaba, Brazil), up to 3 MPa. The pellets were placed in a mold and pressed for 10 min at 180 °C.

For the pressing method, procedures similar to those applied to the pellets from the extrusion process were adopted but using only the homogeneous mixture without passing through the extruder. After the pressing stage, the plates were removed from the press and subjected to a 5-min cooling period at room temperature.

### 2.2. Determination of the Thermal, Physical, and Microstructural Properties of the Composites

The materials were characterized using FTIR analysis. The spectra were obtained with a Bruker spectrophotometer, model Tensor 27 (Billerica, MA, USA), employing the attenuated total reflectance (ATR) technique. Prior to analysis, the samples were ground using a knife mill, resulting in a powdered form. The spectra were recorded in the range of 4000–400 cm^−1^ with a resolution of 4 cm^−1^, and a total of 40 scans were performed.

The X-ray diffraction (XRD) analysis was conducted using a Rigaku^®^ diffractometer, model Miniflex 600 (Rigaku, Tokyo, Japan). The experiment employed a Cu tube with a wavelength of 1.5406 Å, operating at a voltage of 40 kV and a current of 15 mA. The crystallinity analysis of the samples using the XRD technique was performed within the Bragg angle range (2θ) of 10° to 80°, with a step size of 0.02°.

The specimens were placed in the sample holder (with a capacity for six samples) and transferred to the shielded internal compartment of a GreCon Dax 6000 X-ray densitometer. Initially, the equipment was calibrated for continuous scanning operation along the specimen thickness using collimated X-ray beams. The software controls the operation of the X-ray tube, ensuring the generation of a stable beam, which is then collimated by passing through a slit in a metal block. Subsequently, the total X-ray dose is directed onto the sample, with part of the radiation being attenuated. The radiation that passes through the sample is analyzed by a crystal detector, enabling its detection and quantification. The six samples, measuring 5 × 1.5 × 0.14 cm, were placed in the sample holder and sequentially analyzed (irradiated) by the collimated X-ray beam. The scan speed was precisely controlled, allowing the apparent density to be measured at constant intervals along the thickness (averaging 0.14 mm). The sequence of acquisition was as follows: (i) preparation of the specimen, (ii) placement in the sample holder, (iii) initiation of profile reading along the thickness, and (iv) computational output of the densitometric profile. This measurement enables the determination of the average, maximum, and minimum density values. The same samples were used to determine the apparent density by gravimetry using the X-ray densitometer.

The apparent density of the composite specimens was determined by measuring their volume (length, width, and thickness) using a digital caliper with a precision of 0.1 mm and their mass using a digital balance with a precision of 0.001 g.

#### Thermogravimetric Analysis (TGA)

The thermogravimetric analysis provided insights into the influence of charcoal fine additions on the thermal degradation temperature. For this purpose, a LABSYS EVO SETARAM instrument (LabWrench, Midland, ON, Canada) was used. The analysis was conducted under an inert nitrogen atmosphere to prevent oxidation, with a heating rate of 10 °C/min on ground composite samples with a particle size of 60 mesh. Thermograms were recorded at room temperature (25 °C) under a nitrogen gas atmosphere with a flow rate of 1.8 bar, up to 600 °C. The total duration of the analysis was approximately 200 min.

After the flexural test, the fracture region morphology of the specimens from each condition (0, 5, 10, and 15% fine charcoal particles) was evaluated using SEM images. The specimens obtained from the tensile test were cut into 1-cm pieces to fit the sample holder of the equipment. The samples were fixed onto the appropriate sample holder using double-sided carbon conductive tape to establish electrical contact between the sample and the holder, aided by carbon-based paint, and were gold-coated for 10 s. Finally, micrographs were obtained using a JEOL JSM 840A scanning electron microscope (LabWrench, Midland, ON, Canada), operating with an electron beam at an acceleration voltage of 10 kV and a current of 6 × 10^−11^ A.

## 3. Results and Discussion

### 3.1. Fourier Transform Infrared Spectroscopy (FTIR)

**Figure 2:**
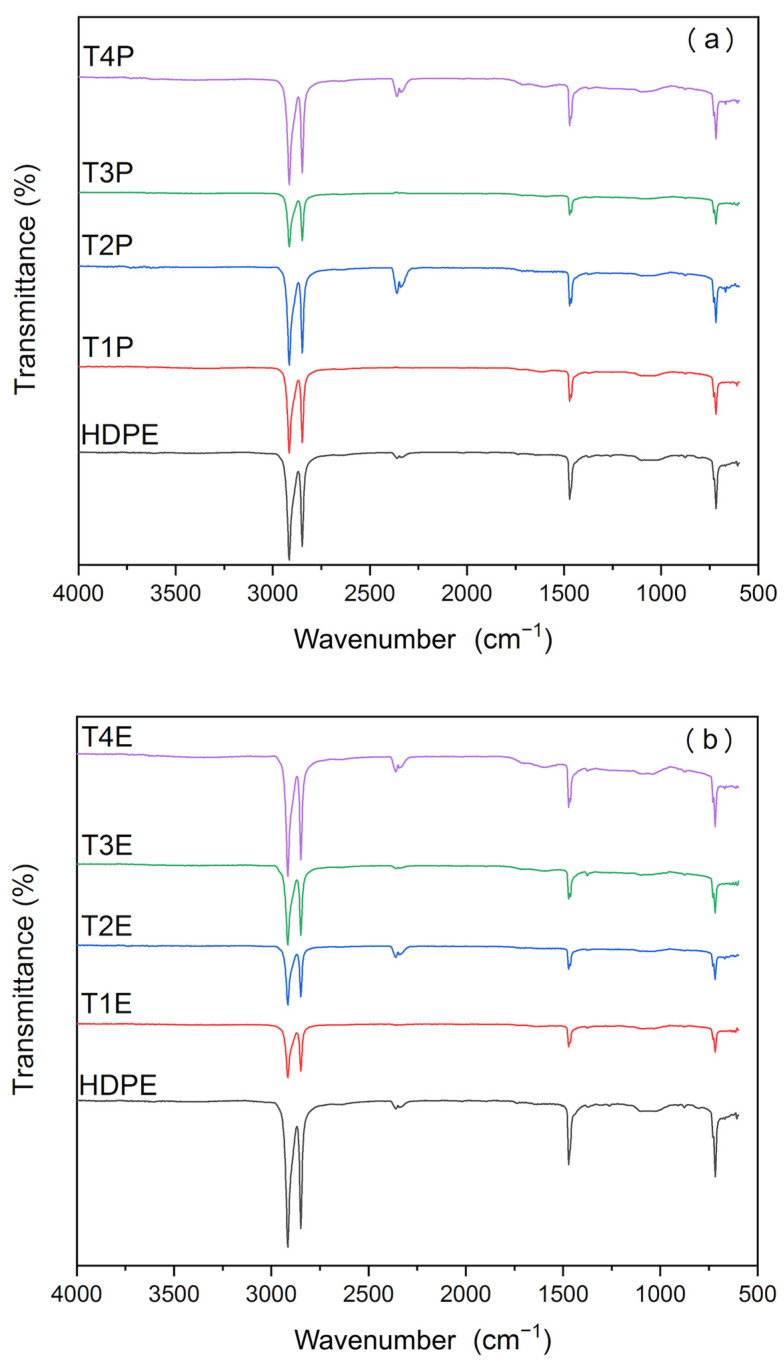
FTIR spectra of the composites produced by the (**a**) pressing and (**b**) extrusion methods.

Figure 2a,b display the FTIR spectra of the composites obtained via the pressing and extrusion methods, respectively. The stretching vibrations of the C-H bond were evidenced by broad bands at 2848 and 2814 cm^−1^. The band at 2360 cm^−1^ corresponds to the asymmetric stretching vibration of the C-H bond in CH_2_ groups, while the band at 716 cm^−1^ represents the out-of-plane in-phase deformation of C-H bonds. Additionally, the bands observed from 670 to 610 cm^−1^ are associated with the angular out-of-plane in-phase deformation of C-H bonds [14].

In the composites obtained through extrusion, a more pronounced shift in the spectral bands was observed, particularly in treatment 4, which involved the incorporation of 15% charcoal fines. The spectra indicated a significant increase in the presence of carbonyl (1800–1600 cm^−1^) and hydroxyl (3100–3700 cm^−1^) groups in the composites compared to the HDPE polymer matrix, as evidenced by the higher intensity of the peaks recorded in the composite with 15% fines.

The analysis performed using the FTIR technique revealed that the incorporation of charcoal fines did not result in detectable interactions with the high-density polyethylene (HDPE) matrix in the composites studied. This conclusion is supported by the maintenance of spectral characteristics similar to the pure matrix, as observed in treatment T1 (without the addition of charcoal fines). Therefore, it is concluded that no significant structural changes occurred between the components that could be detected by this analysis. Similar results were reported by [15,16,17,18], who observed that the FTIR spectra of the composites exhibited characteristic profiles similar to the polymeric matrix, even after the addition of charcoal. These data suggest the absence of significant structural changes in the composites or the predominance of polymer bands in the FTIR spectra, due to the polymer’s higher concentration within the composite.

Although no new absorption bands were observed in the FTIR spectra after the incorporation of charcoal fines, it is important to note that this technique may lack sufficient sensitivity to detect minor or noncovalent interactions at the interface, particularly when the polymer matrix dominates the signal. Thus, the absence of detectable changes in the spectra does not necessarily rule out the occurrence of interfacial interactions. Nevertheless, changes in the intensity of some absorption bands were also recorded, attributed to the incorporation of charcoal into the resin matrix, suggesting possible alterations.

### 3.2. X-Ray Diffraction

**Figure 3:**
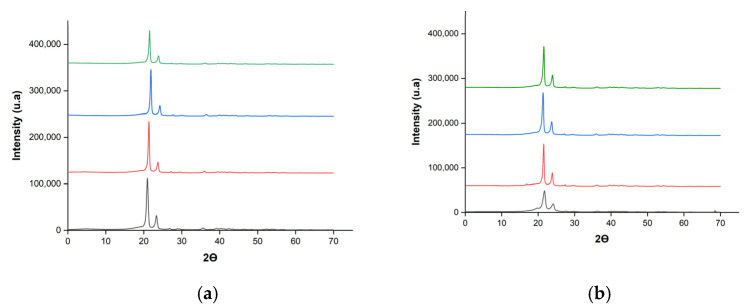
X-ray diffractograms of the composites obtained by the following methods: (**a**) pressing and (**b**) extrusion. The black, red, blue and green lines represent the behavior of materials T1; T2, T3 and T4, respectively.

X-ray diffraction was employed to investigate the modifications in the crystalline structures of the composites produced by pressing and extrusion as a function of the variations in the concentration of coal fines. Figure 3 presents the diffractograms obtained for the composites produced by each method. Figure 3a illustrates the diffractograms of the composites produced by the pressing method, while Figure 3b displays those obtained through the extrusion method.

The X-ray diffraction (XRD) patterns obtained from the analysis exhibited a semi-crystalline structure characterized by two intense diffraction peaks at 2θ = 21.2° and 23.5°, corresponding to the orthorhombic unit cell peaks (110) and (200) of polyethylene [19,20,21]. The coexistence of crystalline phases is a typical characteristic of high-density polyethylene (HDPE). According to [20,21], HDPE exhibits two characteristic peaks at 2θ = 21.2° and 23.5° within the crystalline region of polyethylene, with the most intense peak occurring at 21.2°. The linear and minimally branched structure of HDPE promotes a high degree of crystallinity, leading to greater molecular order [22]. The XDR analysis indicated that the addition of coal fines and the different production methods did not significantly alter the position of these peaks. Therefore, it can be concluded that coal fines do not influence or modify the orthorhombic unit cell of HDPE.

On the other hand, variations in the intensity of the crystallinity peaks were observed as a function of the production method and the concentration of coal fines. Composites produced by pressing generally exhibited more intense peaks, with a slight tendency to decrease as the filler concentration increased. The decrease in the intensity of the X-ray diffraction (XRD) peaks, observed with the increasing load of coal fines using the pressing method, indicates a reduction in the crystallinity of the composites [23,24]. However, the extruded composites did not follow this trend, displaying heterogeneity in the peak intensity. The baseline (background noise) appears higher and more irregular in the second graph, which may suggest a more pronounced amorphous phase present. Charcoal fines had a more significant influence on the crystallinity of the materials produced by extrusion, promoting greater amorphization and disruption of the composite’s crystalline structure. This difference may be associated with the high mechanical shear promoted by the extrusion method, which can break polymer chains and generate disorder in the polymer’s molecular structure, or with the filler dispersion resulting from the different processing methods.

The elevated background noise and less intense diffraction peaks in the XRD patterns of the extruded composites may be partially explained by the mechanical shear and thermal history associated with the extrusion process. Although these samples were later subjected to pressing, which typically favors recrystallization due to slower cooling and applied pressure, the prior exposure to intense shear and thermal stress during extrusion may have led to polymer chain scission or structural rearrangements that hinder subsequent crystallization. In contrast, samples produced solely by pressing, without undergoing prior extrusion, exhibit higher crystallinity, likely due to the preservation of the chain integrity and more favorable conditions for crystal growth.

The relationship between the crystallinity of the HDPE composites and the presence of particles is complex and depends on various factors, such as particle size and concentration. Ref. [25] observed that, at low concentrations of graphene nanoplatelets (GNPs), crystal growth was inhibited, resulting in lower crystallinity of the high-density polyethylene (HDPE) nanocomposite. However, at higher concentrations, particularly with larger-diameter GNPs, crystal growth was enhanced, possibly due to the formation of GNP networks that promote nucleation. This complexity highlights the need for more specific studies on the crystallization kinetics of these composites. Future research should investigate whether charcoal fines act as a nucleating agent for HDPE.

The preservation of the fundamental crystalline structure, even with the addition of fillers and different production processes, indicates high stability of the polymer matrix. The high crystallinity of HDPE imparts superior rigidity, as well as chemical and thermal resistance to the material—essential characteristics for various applications requiring high strength, durability, and resistance to chemical and thermal agents. Moreover, the well-defined crystalline structure contributes to greater dimensional stability, reducing deformation under the load.

Its rigidity, combined with chemical and thermal resistance, along with the lightweight nature and chemical inertness of HDPE, makes it ideal for the production of more durable and resistant packaging, thereby contributing to waste reduction and promoting the circular economy.

Additionally, the presence of fine charcoal particles endows the material with antistatic properties and ultraviolet radiation absorption capabilities, further expanding its potential for applications in various sectors, including the automotive, construction, and agricultural industries. The sustainability of this composite is reinforced using a renewable resource such as charcoal, contributing to the reduction of dependence on fossil-based materials and fostering a more sustainable future.

### 3.3. Thermogravimetric Analysis (TGA)

**Figure 4:**
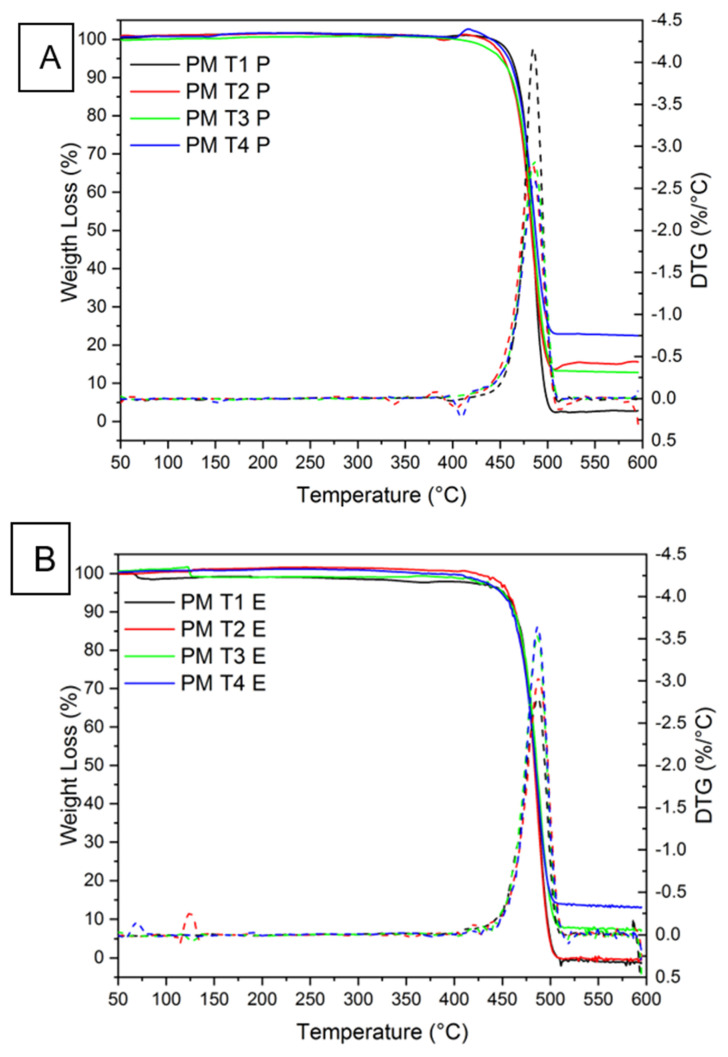
Thermogravimetric (TG, solid lines) and derivative thermogravimetric (DTG, dotted lines) curves of the composites produced by the (**A**) pressing and (**B**) extrusion methods.

The thermogravimetric analysis (TGA) was employed to infer the thermal stability and composition of the composite materials produced by pressing and extrusion, with varying contents of charcoal fines (0, 5, 10, and 15 wt%). The TGA results, presented in Figure 4A,B, provide detailed information on the thermal behavior of the materials, including mass loss and decomposition temperature. Figure 4A displays the TG and DTG curves of the composites produced by the pressing method, while Figure 4B illustrates those made by the extrusion method. Figure 4A,B correspond to the treatments T1 (100% HDPE/0% fines), T2 (95% HDPE/5% fines), T3 (90% HDPE/10% fines), and T4 (85% HDPE/15% fines), respectively.

The thermogravimetric curves for the investigated materials indicate a mass loss profile characterized by two distinct stages. The first stage observed up to 400 °C corresponds to a relatively small mass loss (2–4%), attributed to the evaporation of water adsorbed on the surface of the material or the removal of residual solvents used during the synthesis process. At 400 °C, a single mass loss step is observed, corresponding to the degradation of the polymeric matrix. After this stage, at a temperature of 600 °C, HDPE exhibited different residual amounts, depending on the processing technique employed: 0% to T1E and 3% to T1P. The residue observed for T1P may be associated with contaminants, given that the polymer is recycled. In the case of T1E, the absence of an inorganic filler explains that the complete thermal decomposition during extrusion may have limited the formation of a detectable residue. Furthermore, as indicated by the XRD results, this difference may be related to the high mechanical shear promoted by the extrusion method, which can break polymer chains and generate disorder in the polymer’s molecular structure or filler dispersion resulting from the different processing methods. On the other hand, the thermal behavior of the HPDE, evidenced by thermogravimetric analysis, is similar to that reported in [26]. The composite material developed using birch fiber and high-density polyethylene (HDPE) exhibited excellent thermal stability up to approximately 400 °C, experiencing a mass loss in the temperature range of 400–500 °C, showing a single peak in the first derivative curve with a maximum DTG at 475 °C. The addition of fillers may significantly influence this temperature.

The pressed composites (group P) exhibited higher residual amounts. For T2P and T3P, the residual mass increased to 16%, and for T4P, it reached 22%, reflecting the progressive increase in biochar content (5%, 10%, and 15 wt%, respectively) in the formulations processed by pressing. These results indicate that the biochar is thermally stable under the analysis conditions and remains in the residue after polymer degradation. It is noteworthy that, for the extruded samples with a higher biochar content (T3E and T4E), the residual amounts were 5% and 16%, respectively. Although these values were consistent with the increase in biochar concentration, they were lower than those observed for the corresponding pressed samples. This difference may be related to the effect of the extrusion process, which involves an intense mechanical shear that can influence the dispersion and interaction of the biochar with the polymeric matrix, possibly leading to the partial degradation or removal of some filler particles during processing.

The derivative thermogravimetric (DTG) curves indicated that the maximum decomposition temperature for all the analyzed composites was approximately 487 °C, a value consistent with that reported in the literature for pure high-density polyethylene (HDPE), which is 482 °C [27]. In comparison, Sathish et al. [28] observed a slower decomposition rate for jute fibers, ranging from 205 °C to 265 °C, characterized by gradual mass loss, while a faster decomposition rate occurred between 265 °C and 360 °C. Furthermore, Norizan et al. [29,30] reported TG and DTG curves for oil palm shell powder within the temperature range of 35 °C to 900 °C. It was observed that natural fibers generally exhibit three main stages of thermal degradation, with most of the decomposition process occurring in the range of 215–470 °C [30]. This result demonstrates that the production methods used and the incorporation of charcoal fines did not induce significant changes in the thermal stability of the HDPE matrix. In contrast to the present study, [1] reported that the addition of coal gangue (CG) to polyethylene (PE) in percentages ranging from 10 to 90 wt% could be an effective strategy for enhancing the thermal stability of polymer composites. The formation of a carbonaceous layer on the material’s surface during decomposition acts as a thermal barrier, expanding the potential applications of these materials in high-temperature environments.

The preservation of thermal stability under challenging conditions, such as high temperatures, thermal cycles, aggressive environments, and prolonged heat exposure, demonstrates the feasibility of developing composites with optimized thermal properties. This feature, combined with the potential use of recycled materials like HDPE and bio-reinforcement with charcoal fines, makes these composites a sustainable and efficient alternative for applications in various industrial sectors.

### 3.4. Scanning Electron Microscopy (SEM)

**Figure 5:**
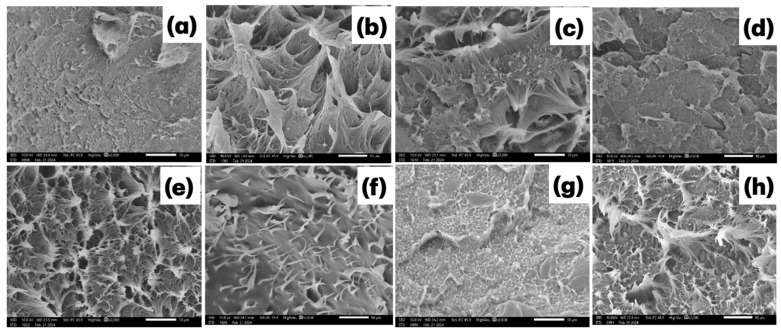
Scanning electron micrographs of HDPE/charcoal fine composites obtained by the pressing (**a**–**d**) and extrusion (**e**–**h**) methods at 2000× magnification. (**a**) T1P-100:0; (**b**) T2P-95:5; (**c**) T3P-90:10; (**d**) T4P-85:15; (**e**) T1E-100:0; (**f**) T2E-95:5; (**g**) T3E-90:10; (**h**) T4E-85:15.

Figure 5 presents SEM imagens of the fracture region of composites containing different proportions of coal fines manufactured using the extrusion method. The imagens a, b, and c correspond to treatments with 0, 5, and 10 wt% coal fines, respectively, and a uniform distribution of coal fine particles within the high-density polyethylene (HDPE) matrix was observed. The incorporation of charcoal fines led to noticeable changes in the morphology. For the pressed samples (Figure 5b–d), the presence of charcoal particles is visible, and an increase in surface roughness and the appearance of voids and pull-outs are evident with the increasing filler content. For the extruded samples (Figure 5f–h), a more compact and continuous morphology is observed compared to the pressed samples, with fewer visible voids and a more uniform distribution of the filler, particularly at lower concentrations (T2E and T3E). However, in T4E (Figure 5h), despite the improved dispersion promoted by the extrusion process, agglomerates and interfacial discontinuities are still evident, indicating that the high filler content limits the efficiency of the dispersion process and affects matrix–filler interactions.

**Figure 6:**
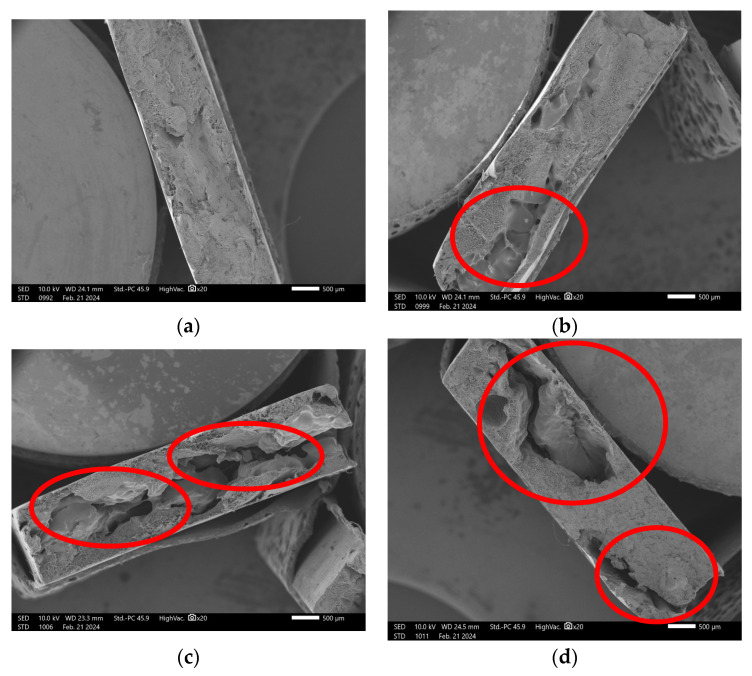
Micrograph of HDPE/charcoal fine composites using the pressing method: (**a**) T1-100:0; (**b**) T2-95:5; (**c**) T3-90:10; (**d**) T4-85:15. Red circles highlight voids formed at the fracture surface.

Figure 6d, highlighted by red circles, reveals an inadequate particle distribution in treatment T4, which contains 15% coal fines. This heterogeneity compromises the reinforcement efficiency of the material. Previous studies, such as that of [31], indicate that smaller particles tend to enhance the interaction between the matrix and the reinforcement. However, this effect was not observed in the present case, possibly due to the broad particle size distribution (0.425 mm > 0.250 mm) and the high particle concentration in the analyzed treatment. The observed phenomenon, highlighted by the red circle and commonly referred to as mechanical anchoring, is characterized by the physical fixation of particles within the matrix without the formation of chemical bonds. The physical interpenetration of the particles with the matrix, as evidenced by the observed detachment, suggests the need to enhance interfacial adhesion in these composites. The incorporation of coupling agents could be an effective strategy to strengthen the interface and improve the performance of the composites. A coupling agent is a chemical compound that acts at the interface to create a chemical bridge between the reinforcement and the matrix. It enhances interfacial adhesion when one end of the molecule is anchored to the surface of the reinforcement while the functional group at the other end reacts with the polymer phase [32]. Future studies should explore the use of compatibilizers or the surface modification of charcoal particles to enhance interfacial adhesion. The incorporation of coupling agents, such as maleic anhydride-functionalized polyethylene, could mitigate interfacial discontinuities and improve filler dispersion, particularly at higher loadings, as already demonstrated in similar polymeric systems.

The composites manufactured using the pressing method exhibited distinct fracture surface morphologies across the different formulations tested. The composite containing 100% HDPE (Figure 5a) displayed a smooth fracture surface, whereas the composites incorporating 5% and 10% charcoal fines (Figure 5b, c) presented a striated and rough morphology, characterized by deep valleys and some coarse ridges, where the stretching of the plastic material was clearly visible. However, in the composite with 15% charcoal fines (Figure 5d), mechanical anchoring of the particles within the matrix was observed, as indicated by the yellow circle, a behavior similar to that seen in the materials produced via the extrusion process.

**Figure 7:**
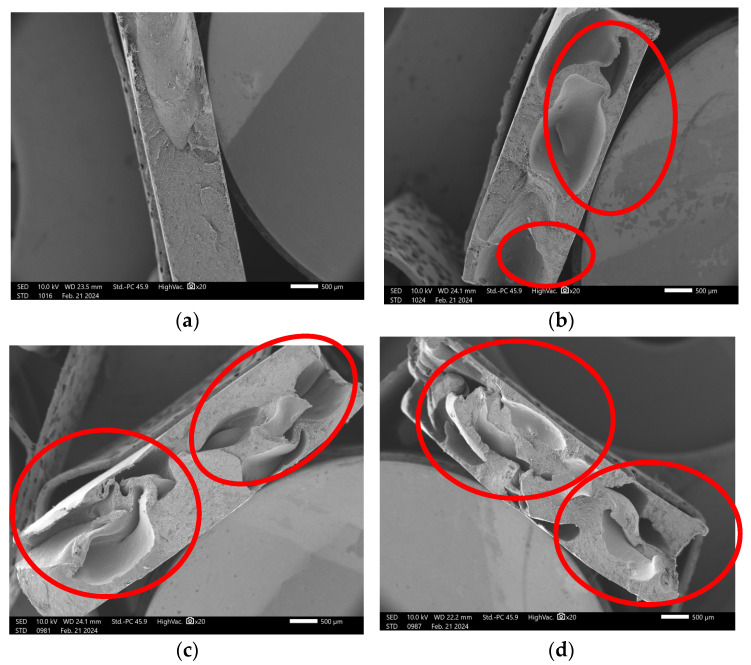
Micrograph of HDPE/charcoal fine composites using the extrusion method: (**a**) T1-100:0; (**b**) T2-95:5; (**c**) T3-90:10; (**d**) T4-85:15. Red circles highlight voids formed at the fracture surface.

Figure 6 and Figure 7 present SEM imagens of the fracture surfaces of composites containing 0%, 5%, 10%, and 15% charcoal fines. Figure 6 illustrates the fractures of composites manufactured using the pressing method, while Figure 7 corresponds to those produced via the extrusion method. Figure 6a and Figure 7a show the fracture imagens in the absence of charcoal fines, revealing a smooth surface characterized by a lack of cracks or evident irregularities. In contrast, Figure 6b–d and Figure 7b–d—corresponding to treatments T2, T3, and T4 containing 5%, 10%, and 15% fines, respectively—demonstrate an increasing presence of voids (highlighted by red circles) as the concentration of charcoal fines increases, regardless of the manufacturing method employed.

According to [33], the presence of voids in the polymer matrix at concentrations exceeding 20% of the total volume significantly compromises the mechanical properties of composite materials. Although porosity is an intrinsic characteristic of carbonaceous materials, such as charcoal, and is often associated with inferior mechanical properties, it is a crucial feature for the production of biomaterials. The ability to control the porosity of these materials allows for the adjustment of their properties to promote cell adhesion, proliferation, and differentiation, making them promising candidates for tissue engineering.

The great potential for various applications, ranging from filtration (air and water) to tissue engineering, and the versatility of these materials stem from the combination of properties such as high porosity, low density, electrical conductivity, and adsorption capacity. However, the material’s performance in a given application will depend on the optimization of its properties to meet the specific requirements of each case.

In this context, the flexibility in customizing HDPE and charcoal composites allows them to be adapted for diverse applications. The variation in the charcoal content and the control of porosity enables the adjustment of material properties according to specific needs. Additionally, the use of charcoal as a bio-reinforcement contributes to the sustainability of the production process by promoting the use of a renewable resource.

### 3.5. X-Ray Densitometry

**Figure 8:**
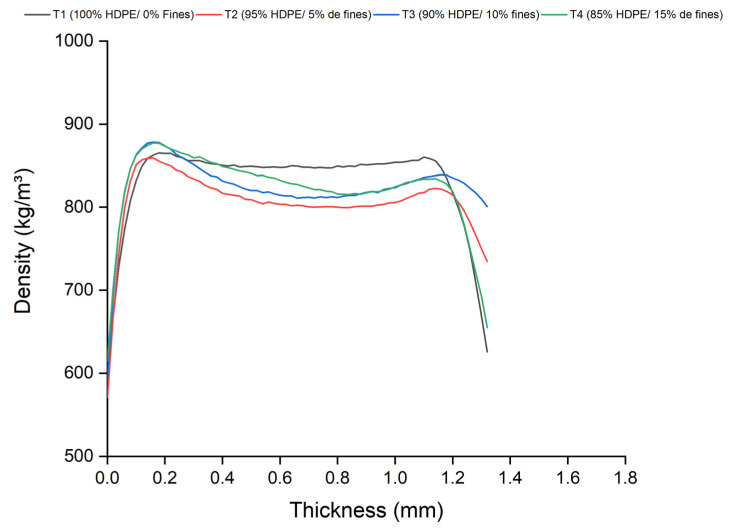
Average apparent density profile of the composites produced by the pressing method.

The density profile, as defined by [34], represents the distribution of density along the thickness of the composite. This analysis is essential for identifying density heterogeneities, particularly in the central region, which may be attributed to the raw material characteristics and the manufacturing process. The curves presented in Figure 8 illustrate the density profile obtained from the average densities measured for each of the four treatments applied using the pressing method. This analysis enables the comparison of the influence of physical, chemical, mechanical, and thermal variables on the material’s behavior.

The profile indicated a trend toward density homogenization in the central region of the pressed composites, particularly in the treatments involving the addition of fine particles. This finding suggests that an increase in the concentration of fine particles did not result in significant variations along the composite’s thickness. The analysis of the pressed composites revealed a characteristic “M”-shaped density profile. This “M”-shaped profile is defined by high-density surfaces at the edges and a lower-density center, a pattern typically observed in hot-pressed composites [35,36]. The higher density at the edges and the lower density at the center may cause variations in the distribution of mechanical stresses, impacting the overall strength of the material. The center with lower density is more prone to the initiation of cracks and fractures, compromising the composite’s service life. This less dense region can act as a weak point, reducing the material’s mechanical strength. This configuration is associated with the pressing process and the internal pressure distribution during its execution, as the lateral pressure is higher than the internal pressure, resulting in this pressure gradient.

**Figure 9:**
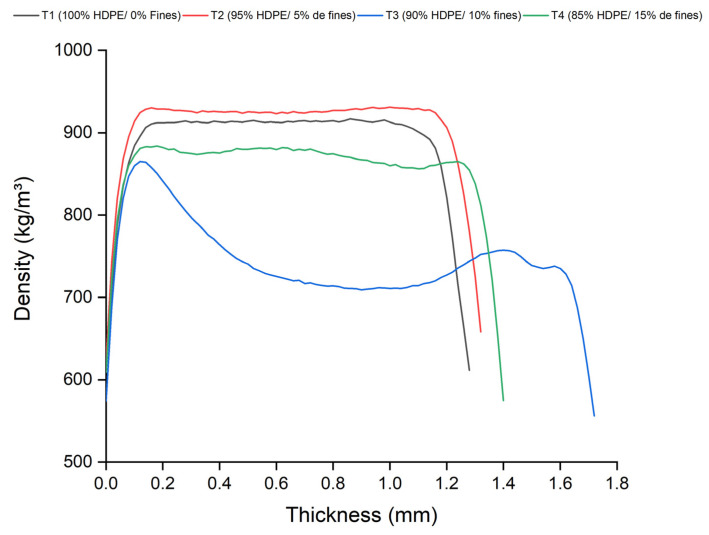
Average apparent density profile of the composites produced by the extrusion method.

The composites produced by extrusion exhibited density profiles with greater variation among the treatments, as evidenced in Figure 9. Treatment 3, composed of 90% HDPE and 10% fines, displayed an atypical behavior, distinguishing itself from the others. The significant difference observed in the density profile of treatment 3 compared to the other treatments suggests the occurrence of failures in the production process, such as the heterogeneous distribution of the fines. This structural non-uniformity may result from difficulty in dispersing the fines within the polymer matrix, compromising the quality of the final material.

**Figure 10:**
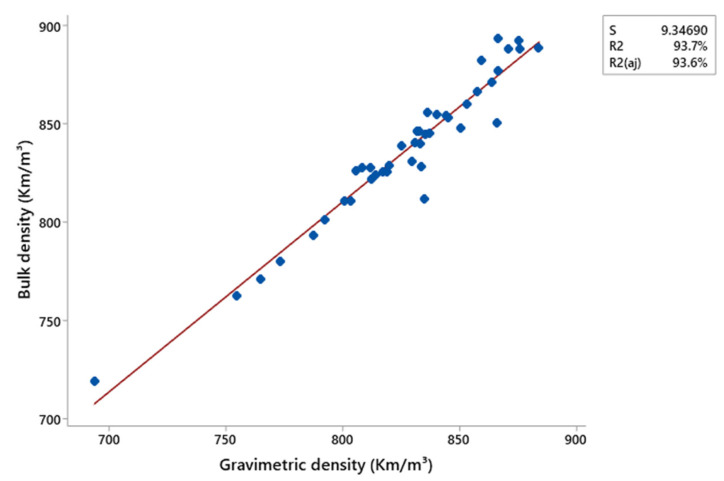
Correlation of the average apparent density of the composites produced by the pressing method through X-ray densitometry and gravimetry.

**Figure 11:**
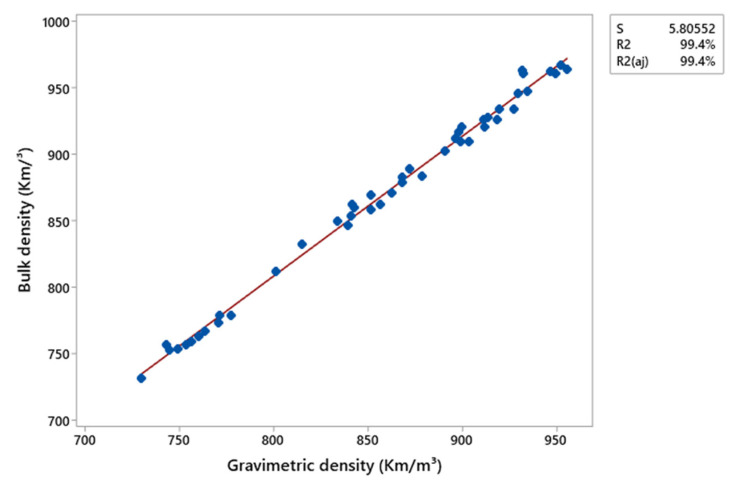
Correlation of the average apparent density of the composites produced by the extrusion method through X-ray densitometry and gravimetry.

The X-ray densitometry profiles indicate that the production method influenced the structural characteristics of the composites. Pressing promotes the formation of more homogeneous profiles, while extrusion results in higher average densities with greater variation, which can be attributed to the increased compaction of the material during the manufacturing process or partial degradation of the polymeric chains. Conversely, lower compaction results in higher porosity and reduced stiffness [37]. In this context, the addition of charcoal fines may compromise the mechanical properties of the composites due to the observed reduction in density. Thus, the composites produced by pressing possibly exhibited superior mechanical performance due to their more homogeneous density profiles. The comparison between the average apparent density obtained by X-ray densitometry and the gravimetric density, presented in Figure 10 and Figure 11, revealed a strong linear correlation for the composites produced by both methods, pressing and extrusion. The determination coefficients (R^2^) of 93.7% and 99.4% for the pressing and extrusion methods, respectively, indicate an excellent agreement between the results and confirm the reliability of X-ray densitometry for determining the density of these materials.

The results of this study highlight the use of X-ray densitometry as an effective tool for characterizing density and density distribution in a wide range of composites, demonstrating its applicability in the non-destructive analysis of these materials.

## 4. Conclusions

- The production methods by pressing and extrusion, as well as the addition of charcoal fines, did not exhibit a significant influence on the thermal properties and crystallinity of the investigated composites.
- The microstructural analysis, conducted through scanning electron microscopy (SEM), confirmed an increase in the porosity of the composites, which may explain the reduction in mechanical properties observed during the tensile testing and non-destructive methods.
- The analysis by Fourier transform infrared (FTIR) spectroscopy did not reveal significant differences with the addition of charcoal fines, as no additional peaks were observed in the spectrum. These results suggest that the charcoal fines acted as a bio-reinforcement, exerting a stabilizing or conservating effect on the material’s structure.
- The thermogravimetric analysis (TGA) and X-ray diffraction (XRD) demonstrated that the addition of fine charcoal particles to high-density polyethylene (HDPE), regardless of the processing method, did not significantly affect the thermal stability or crystallinity of the composites. These characteristics indicate a high potential for industrial applications requiring thermal resistance and dimensional stability.
- The composites exhibited potential for applications that demand low weight, thermal stability, and high porosity. Additionally, these materials contribute to sustainability by valorizing plastic waste and promoting the circular economy.
- The use of fine charcoal particles as a filler for high-density polyethylene (HDPE) presents significant potential, offering advantages such as waste mitigation, the valorization of byproducts, and the possibility of developing materials with enhanced properties.

## Figures and Tables

**Table 1:** Experimental design for the manufacture of composites.

Production Process	Treatments	HDPE (%)	Fines (%)
Pressing	T1	100	0
	T2	95	5
	T3	90	10
	T4	85	15
Extrusion	T1	100	0
	T2	95	5
	T3	90	10
	T4	85	15

## Data Availability

The original contributions presented in this study are included in the article. Further inquiries can be directed to the corresponding author(s).

## Abbreviations

The following abbreviations are used in this manuscript:

AE	Statistical Clustering
CEFET	Federal Center for Technological Education
XRD	X-ray Diffraction
FTIR	Fourier Transform Infrared Spectroscopy
SEM	Scanning Electron Microscopy
PE	Polyethylene
HDPE	High-Density Polyethylene
TGA	Thermogravimetric Analysis
